# Alcohol enhances type 1 interferon-α production and mortality in young mice infected with *Mycobacterium tuberculosis*

**DOI:** 10.1371/journal.ppat.1007174

**Published:** 2018-08-02

**Authors:** Deepak Tripathi, Elwyn Welch, Satyanarayana Swamy Cheekatla, Rajesh Kumar Radhakrishnan, Sambasivan Venkatasubramanian, Padmaja Paidipally, Abhinav Van, Buka Samten, Kamakshi P. Devalraju, Venkata Sanjeev Kumar Neela, Vijaya Lakshmi Valluri, Carol Mason, Steve Nelson, Ramakrishna Vankayalapati

**Affiliations:** 1 Department of Pulmonary Immunology, Center for Biomedical Research, University of Texas Health Science Center at Tyler, Tyler, Texas, United States of America; 2 Bhagwan Mahavir Medical Research Center, Hyderabad, India; 3 Department of Medicine, Section of Pulmonary/Critical Care Medicine, Louisiana State University Health Sciences Center, New Orleans, Louisiana, United States of America; Portland VA Medical Center, Oregon Health and Science University, UNITED STATES

## Abstract

In the current study, we used a mouse model and human blood samples to determine the effects of chronic alcohol consumption on immune responses during *Mycobacterium tuberculosis* (*Mtb*) infection. Alcohol increased the mortality of young mice but not old mice with *Mtb* infection. CD11b+Ly6G+ cells are the major source of IFN-α in the lungs of *Mtb*-infected alcohol-fed young mice, and IFN-α enhances macrophage necroptosis in the lungs. Treatment with an anti-IFNAR-1 antibody enhanced the survival of *Mtb*-infected alcohol-fed young mice. In response to *Mtb*, peripheral blood mononuclear cells (PBMCs) from alcoholic young healthy individuals with latent tuberculosis infection (LTBI) produced significantly higher amounts of IFN-α than those from non-alcoholic young healthy LTBI+ individuals and alcoholic and non-alcoholic old healthy LTBI+ individuals. Our study demonstrates that alcohol enhances IFN-α production by CD11b+Ly6G+ cells in the lungs of young *Mtb*-infected mice, which leads to macrophage necroptosis and increased mortality. Our findings also suggest that young alcoholic LTBI+ individuals have a higher risk of developing active TB infection.

## Author summary

Chronic alcohol consumption modulates the host immune defense mechanism(s) and makes the host susceptible to various fungal, viral and bacterial infections, including *Mycobacterium tuberculosis* (*Mtb*). However, limited information is available about the mechanisms involved in alcohol-mediated host susceptibility to *Mtb* and other intracellular bacterial infections. In the current study, we fed control and alcohol diets to young and old mice and determined the mortality rates and the immune mechanisms involved in host susceptibility to *Mtb* infection. We found that alcohol increases the mortality of young mice but not old mice infected with *Mtb*. The increased mortality in alcohol-fed *Mtb*-infected young mice was due to IFN-α production by CD11b+Ly6G+ cells. We also found that PBMCs from young alcoholic individuals with latent tuberculosis infection (LTBI) produced significantly higher amounts of IFN-α than those from young non-alcoholic, old alcoholic and old non-alcoholic LTBI+ individuals. Our findings suggest that young alcoholic LTBI+ individuals have a higher risk of developing active TB infection. Our studies provide the first evidence that chronic alcohol consumption induces IFN-α production in young *Mtb*-infected mice and increases their mortality rates. Further characterization of CD11b+Ly6G+ cells and delineation of the mechanisms through which alcohol enhances IFN-α production in Ly6G+ cells during *Mtb* infection will facilitate the development of therapies for alcoholic individuals with latent and active *Mtb*.

## Introduction

It is estimated that more than two billion people worldwide are infected with *Mycobacterium tuberculosis* (*Mtb*), but only 5–10% of these individuals develop TB during their lifetime [1,2]. The geriatric population represents a large reservoir of latent tuberculosis infection (LTBI) [3]. It is difficult to diagnose and treat tuberculosis in aged individuals [3,4]. Approximately 57% of tuberculosis deaths occur in the aged population (above 50), and this burden is high in developed countries [5]. Immunosuppressive conditions, such as HIV infection, diabetes mellitus and drug and alcohol abuse, are risk factors that increase the chances of tuberculosis (TB) reactivation in people with LTBI [6–9]. In addition, individuals with alcoholism show higher relapse rates and a higher probability of having multidrug-resistant TB [10].

Alcoholism leads to the development of liver cirrhosis, cancer, insulin resistance, epilepsy, hypertension, psoriasis, preterm birth complications, cardiovascular diseases and stroke [11,12]. Chronic alcohol consumption impairs the host immune response to cancer and infections [13]. Alcohol impairs monocyte phagocytic and antigen-presenting capacities and suppresses the alveolar macrophage production of monokines, such as IL-23, in response to infection [14–16]. Alcohol-exposed dendritic cells produce more IL-10 and less IL-12, suggesting an inhibitory effect on dendritic cell function [17,18]. In humans and experimental mice, chronic alcohol consumption makes neutrophils hypo-responsive to bacterial infections[19]. Prolonged alcohol consumption induces type I interferon (IFN) and tumor necrosis factor alpha (TNF alpha) production [20]. Alcohol impairs NK cell trafficking and inhibits NK cell cytotoxicity [21]. Chronic alcohol consumption impairs adaptive immune responses mediated by B and T-cells [22]. These immunosuppressive effects of alcohol are more severe in elderly individuals than in young individuals [23].

Chronic alcohol consumption makes the host susceptible to various bacterial infections, including TB [24]. Epidemiological and immunological evidence strongly suggest a link between alcoholism and the worsening of TB disease [25]. Chronic alcohol consumption impairs the immune responses of *Mtb*-infected mice [19]. Alcohol feeding before BCG vaccination reduces T cell responses, but there are no effects when BCG vaccination is delivered prior to alcohol feeding [26]. These studies were performed after the short term-feeding of mice with alcohol, and the mechanism(s) involved in host susceptibility remain unknown. The long-term effects of alcohol consumption on host defense mechanisms against *Mtb* infection are also unknown, particularly in old individuals.

In this study, we determined the survival of alcohol-fed young and old mice infected with *Mtb*. We also determined the immune mechanisms responsible for the early death of alcohol diet-fed young mice infected with *Mtb*.

## Results

### Young alcoholic mice are susceptible to *Mtb* infection

**Fig 1:**
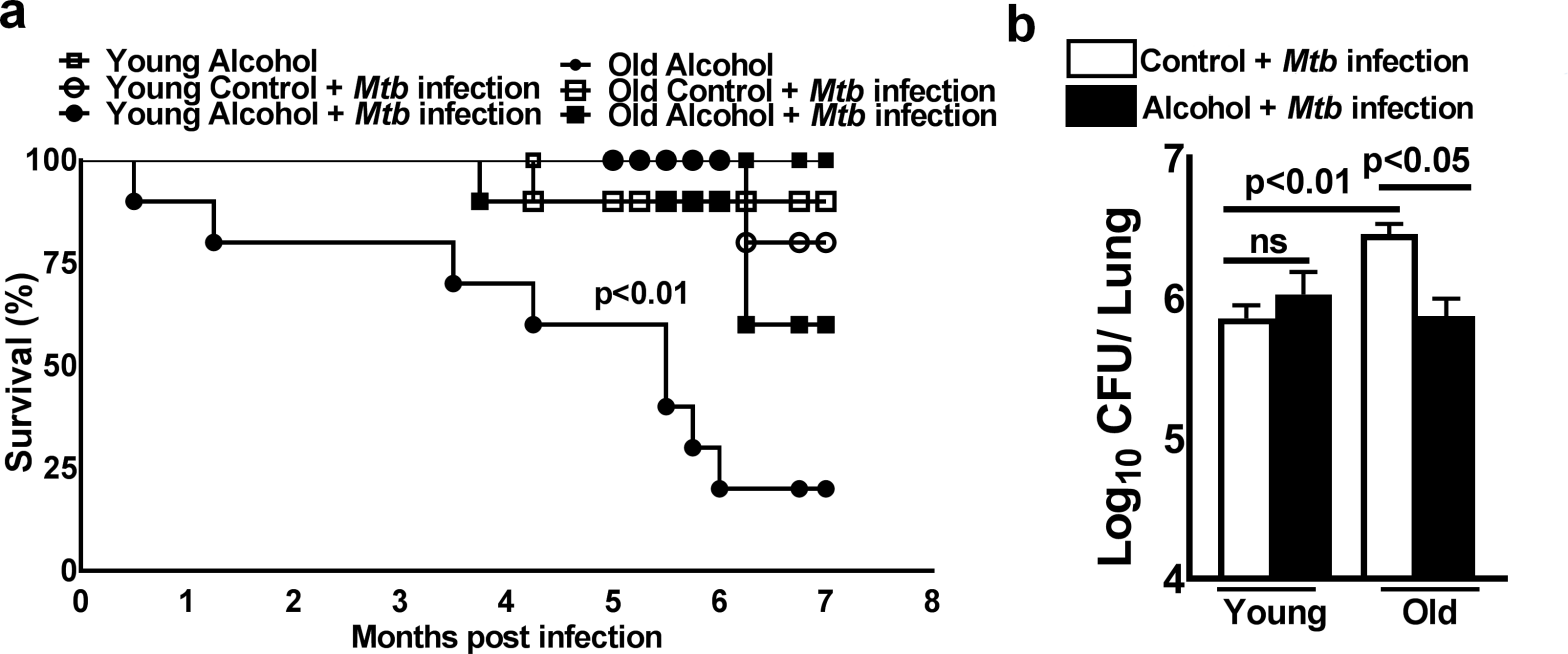
Young alcoholic mice are susceptible to *Mtb* infection. Young (one to two months of age) and old (17 to 22 months of age) mice were fed control and alcohol diets for one month as detailed in the methods section; then, they were infected with 50–100 CFU of aerosolized *Mtb* H37Rv, and control and alcohol diet feeding was continued. **a.** Survival was determined. Survival curves were compared using the log-rank test (P<0.05). **b.** Bacterial burden in lungs at 3 months p.i. The data are representative of two pooled independent experiments (5 mice per group were used for each independent experiment). The mean values, p-values and SEs are shown.

Young and old mice were fed alcohol or control diets for one month and then infected with *Mtb* H37Rv as detailed in the methods section. Alcohol or control diet feeding was continued until the death of the mice or the termination of the experiment. As shown in Fig 1, eighty percent of *Mtb*-infected alcohol-fed young mice died within 6 months (p<0.01, Fig 1A); there was a twenty-five percent death rate in *Mtb*-infected alcohol-fed old mice, a twenty-five percent death rate in *Mtb*-infected control diet-fed old mice and no deaths in the control diet-fed young mice. In these groups of mice, most of the deaths occurred after three months. The bacterial burden in the lungs of these mice was measured three months after *Mtb* infection. As shown in Fig 1B, there was a marginal increase in the bacterial burden in *Mtb*-infected control diet-fed old mice compared to that in the other groups, and there was a marginal but significant decrease in the bacterial burden in the lungs of alcohol-fed old mice. The above results demonstrate that there is no correlation between the bacterial burden and increased mortality in alcoholic mice infected with *Mtb*. Serum alcohol levels and liver alanine transaminase activity were similar among all groups of mice (Figs 1A, 1B and S1).

### Alcohol enhances IFN-*α* production in young mice infected with *Mtb*

**Fig 2:**
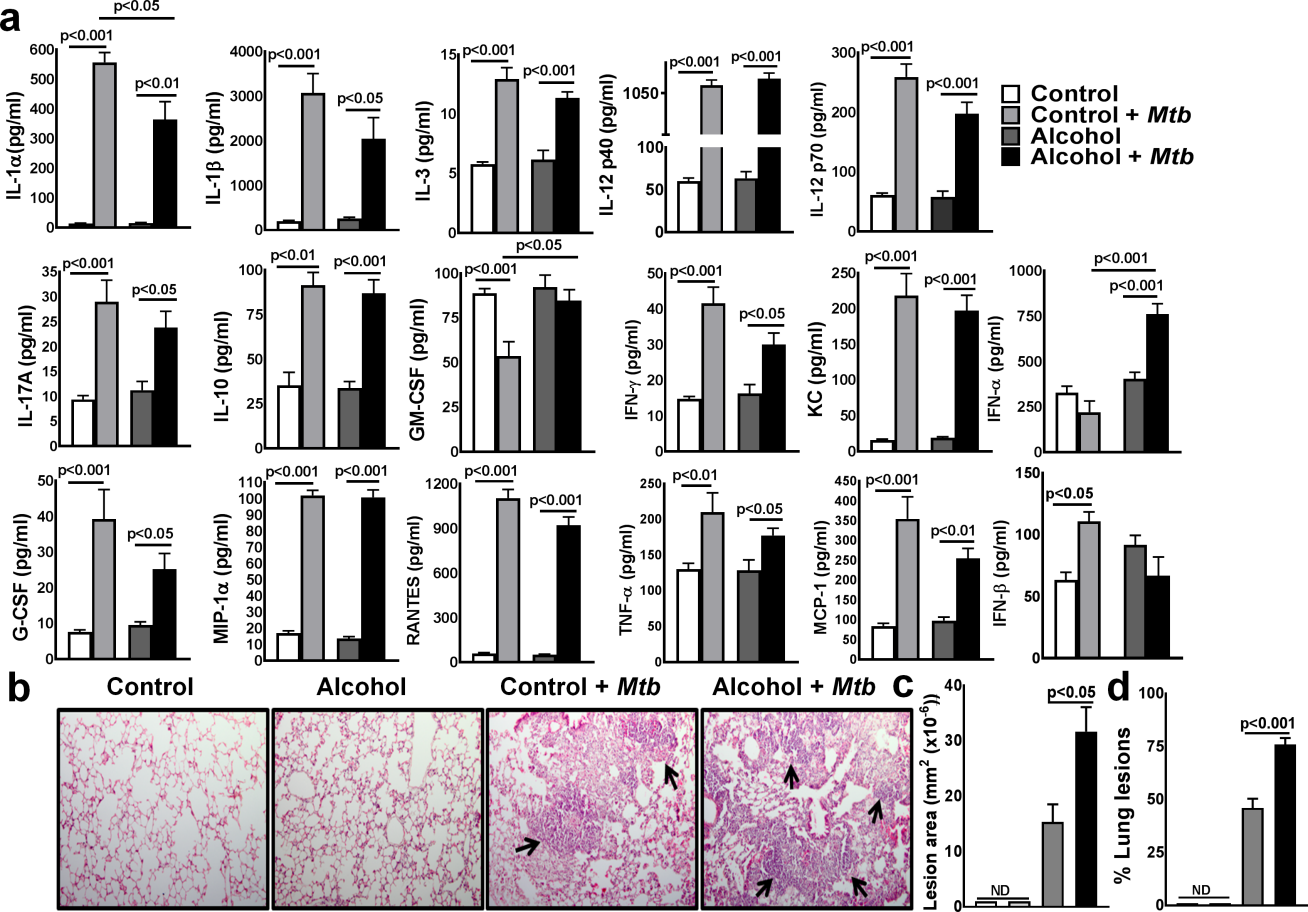
Alcohol enhances IFN-α production in young mice infected with *Mtb*. Young mice were fed control and alcohol diets as detailed in the methods section; then, they were infected with 50–100 CFU of aerosolized *Mtb* H37Rv, and control and alcohol diet feeding was continued. **a.** At three months p.i., lung homogenates from uninfected control and alcohol diet-fed and *Mtb*-infected control and alcohol diet-fed mice were collected, and cytokine and chemokine levels were determined by multiplex ELISA. **b-d.** At three months p.i., lungs from uninfected control and alcohol diet-fed mice as well as from *Mtb*-infected control and *Mtb*-infected alcohol diet-fed mice were isolated and formalin fixed. Paraffin-embedded tissue sections were prepared, and hematoxylin and eosin staining was performed. Inflamed lung areas were compared between the groups. **b.** A representative figure is shown. **c.** The lesion area and **d**. % lung lesions were calculated. The data are representative of two independent experiments. Five mice per group were used for each independent experiment. The mean values, p-values and SEs are shown.

We determined whether alcohol had any effect on the pro- and anti-inflammatory responses of young mice following *Mtb* infection. Young mice were fed control and alcohol diets and infected with *Mtb* as in Fig 1. After three months, the levels of various cytokines and chemokines were measured in the lung homogenates by multiplex (23-plex) ELISA. As shown in Fig 2A, at three months p.i., various cytokines and chemokines were measured, but only IFN-α levels were increased significantly in *Mtb*-infected alcohol-fed young mice compared to those in uninfected alcohol-fed and *Mtb*-infected control diet-fed mice (Fig 2A). There was a marginal but significant decrease in IL-1*α* levels in *Mtb*-infected alcohol-fed young mice compared to those in *Mtb*-infected control diet-fed mice (Fig 2A).

Histological analyses indicated that the number of lesions throughout the lungs was significantly higher in *Mtb*-infected alcohol diet-fed young mice than in *Mtb*-infected young control mice and uninfected young mice (Fig 2B, 2C and 2D).

### CD11b+Ly6G+ cells are the major source of IFN-*α* in *Mtb*-infected young alcoholic mice

**Fig 3:**
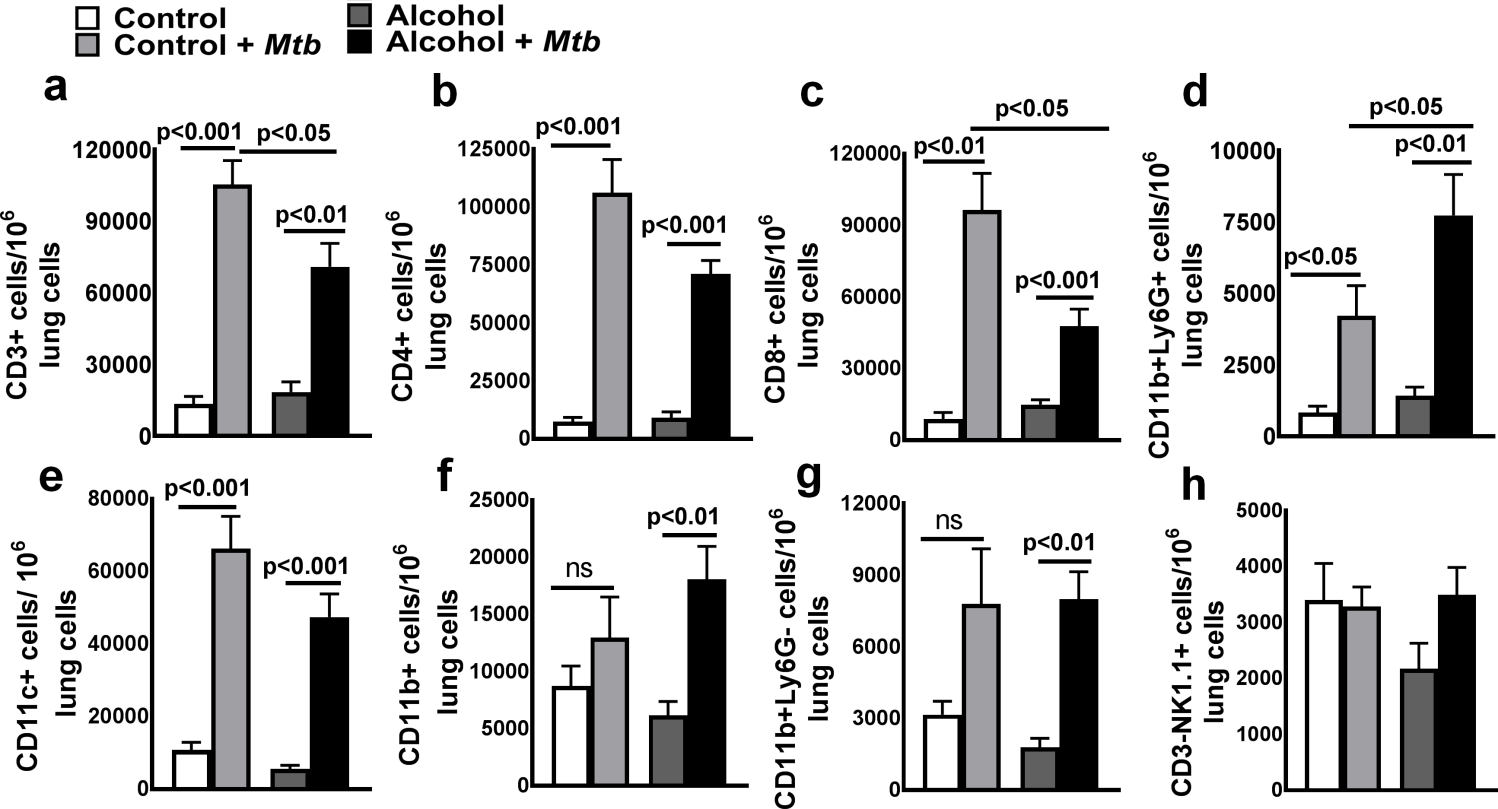
Absolute number of lung leukocyte populations in *Mtb*-infected alcohol and control diet-fed mice. Young control and alcohol diet-fed mice were infected with 50–100 CFU of aerosolized *Mtb*. At three months p.i., lungs from uninfected control and alcohol diet-fed mice and from *Mtb*-infected control and alcohol diet-fed mice were isolated. The absolute numbers of various leukocyte populations, namely, **a**. CD3+ **b**. CD4+ **c**. CD8+ **d**. CD11b+Ly6G+ **e**. CD11c+ **f**. CD11b+ **g**. CD11b+Ly6G- and **h**. CD3-NK1.1+ cells, per 10^6^ total lung cells were determined by flow cytometry. The data are representative of two independent experiments. Five mice per group were used for each independent experiment. The mean values, p-values and SEs are shown.

**Fig 4:**
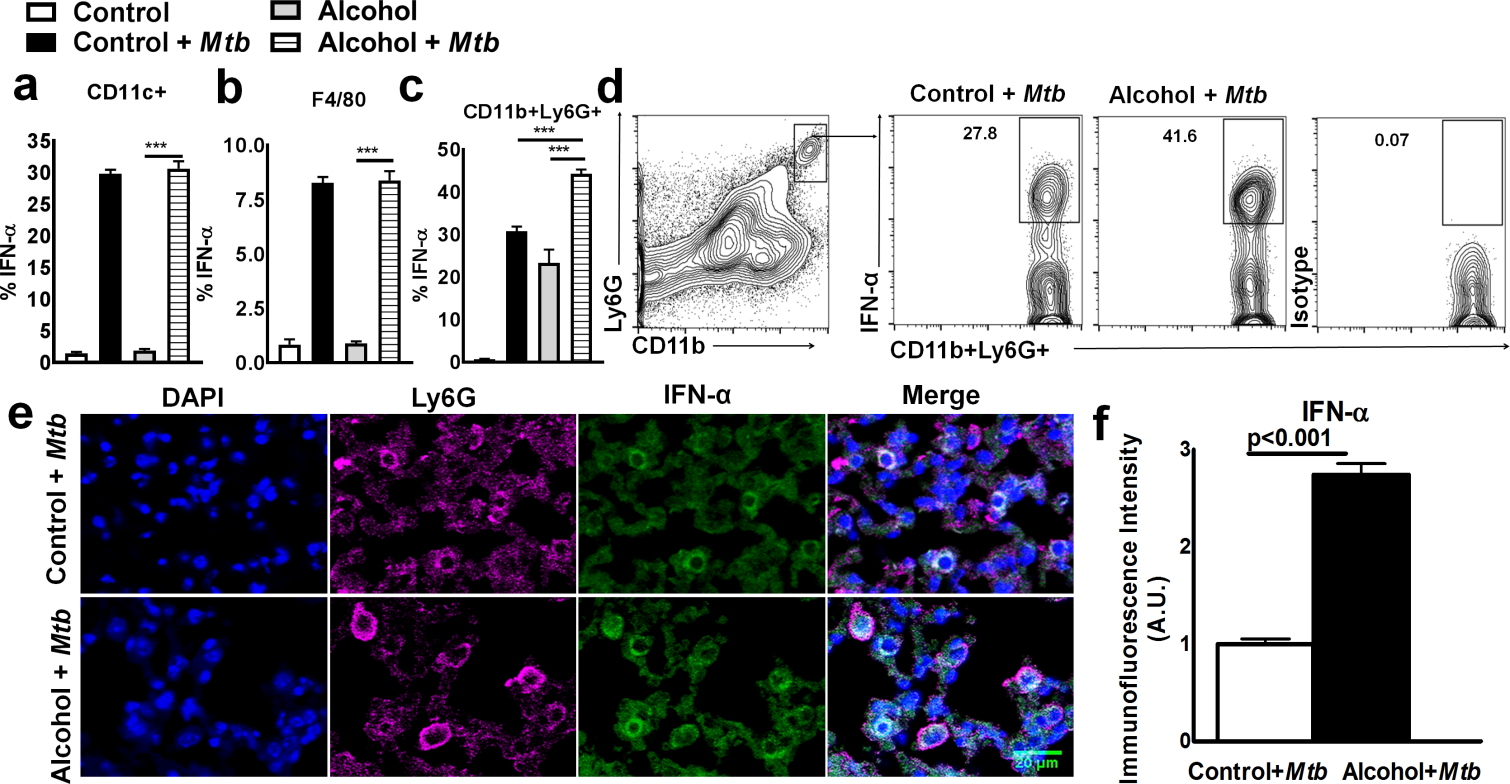
CD11b+Ly6G+ cells are the major source of IFN-α in *Mtb*-infected young alcoholic mice. Young control and alcohol diet-fed mice were infected with 50–100 CFU of aerosolized *Mtb*. At three months p.i., lungs from uninfected control and alcohol diet-fed mice and from *Mtb*-infected control and alcohol diet-fed mice were isolated. The percentages of **a.** IFN-α+CD11c+, **b.** IFN-α+F4/80+ and **c.** IFN-α+CD11b+Ly6G+ cells were determined by flow cytometry. The data are representative of two independent experiments. **d**. A representative flow cytometry figure is shown. **e and f.** At three months p.i., lungs from uninfected control and alcohol diet-fed mice as well as from *Mtb*-infected control and *Mtb*-infected alcohol diet-fed mice were isolated and formalin fixed. Paraffin-embedded tissue sections were prepared and examined for IFN-α+ cells by confocal microscopy. **e.** A representative figure is shown. **f.** The immunofluorescence intensities (AU) of IFN-α were calculated for these groups and are shown. Five mice per group were used for each independent experiment. The mean values, p-values and SEs are shown.

To determine the cellular source of IFN-α in *Mtb-*infected alcohol diet-fed young mice, we first quantified the leukocyte populations by flow cytometry. As shown in Fig 3 and S2A and S2B Fig, at three months after *Mtb* infection, the number of CD11b+Ly6G+ cells in the lungs was significantly higher in *Mtb*-infected alcohol diet-fed young mice than in *Mtb*-infected control mice and uninfected alcohol diet-fed mice. We next determined the phenotype of IFN-α-producing pulmonary cells three months p.i.; there were no significant differences in the absolute numbers of IFN-α-producing CD11c+ and F4/80 cells (Fig 4A and 4B). However, the absolute number of IFN-α-producing CD11b+Ly6G*+* cells in the lungs was significantly higher in *Mtb*-infected young alcoholic mice than in uninfected alcohol diet-fed mice and *Mtb*-infected control mice (Fig 4C and 4D). To confirm our findings that IFN-α levels were increased in the lungs of *Mtb-*infected young alcohol diet-fed mice, mice were euthanized three months p.i., and lung sections were examined for IFN-α+ cells by immunofluorescence staining. As shown in Fig 4F, the mean immunofluorescence intensity for IFN-α was significantly higher in *Mtb*-infected alcohol diet-fed young mice than in *Mtb*-infected control and uninfected alcoholic mice. We also found that Ly6G*+* cells are the major source of IFN-α (Fig 4E). We further characterized this cell population in the lung tissues of *Mtb*-infected young alcohol diet-fed mice. As shown in S6 Fig, Ly6G+IFN-α+ cells were positive for CD11b, CD200 and CD163 but negative for F4/80, CD68, CD115, CD11c, and Ly6C.

### IFN-*α* reduces the survival of *Mtb*-infected alcohol diet-fed young mice

**Fig 5:**
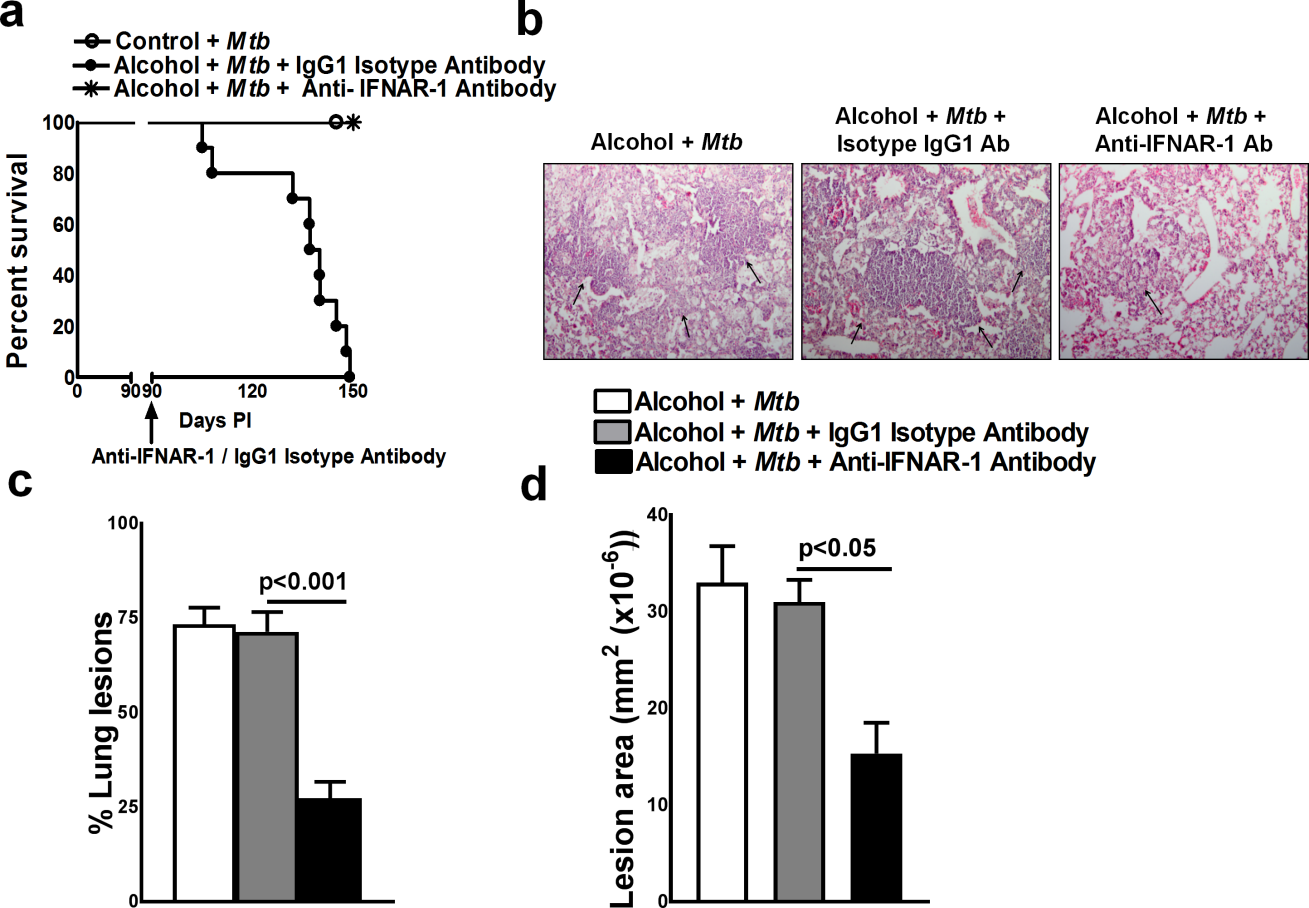
IFN-α reduces the survival of *Mtb*-infected alcohol diet-fed young mice. Control and alcohol diet-fed young mice were infected with 50–100 CFU of aerosolized *Mtb*. At three months p.i., some of the alcohol-fed *Mtb*-infected young mice were treated with either an anti-IFNAR-1 mAb or IgG1 isotype-matched control mAb (0.3 mg per mouse, starting 3 months p.i. every 4 days for 3 months). **a.** Survival rates of *Mtb*-infected alcohol-fed young mice treated with the anti-IFNAR-1 mAb or IgG1 isotype-matched control mAb. The data from two independent experiments were pooled. Five mice per group were used for each independent experiment. The survival curves were compared using the log-rank test (P<0.001). **b to d.** At three months p.i., *Mtb*-infected alcohol diet-fed mice were treated with either the anti-IFNAR-1 mAb or IgG1 isotype-matched control mAb. Lungs were isolated and formalin-fixed. Paraffin-embedded tissue sections were prepared, and hematoxylin and eosin staining was performed. **b.** A representative figure is shown. **c.** The % lung lesions and **d.** lesion area were calculated. The data from two independent experiments were pooled. Three mice per group were used for each independent experiment (n = 6). The mean values, p-values and SEs are shown.

Type 1 interferons have a protective role in viral infections [27]. However, in *Mtb* infection, type 1 interferon signaling causes immunopathology and early mortality in the infected mice [28]. We investigated whether the increased mortality of *Mtb*-infected alcohol-fed young mice was due to enhanced IFN-α production. Young mice were fed control and alcohol diets and infected with *Mtb* as in Fig 1. After three months, the mice were treated with either a neutralizing anti-IFNAR-1 mAb or an isotype-matched IgG1 control mAb. As shown in Fig 5A, 100% percent (p = 0.001) of the *Mtb*-infected alcohol diet-fed young mice that received the isotype-matched control mAb died within 2 months. In contrast, all mice that received the anti-IFNAR-1 mAb survived. Histological analyses indicated that there were significantly fewer necrotic lesions throughout the lungs of the anti-IFNAR-1 mAb-treated *Mtb*-infected alcohol diet-fed young mice than in those of the isotype antibody-treated *Mtb*-infected alcohol diet-fed young mice (Fig 5B, 5C and 5D).

### IFN-*α* production in *Mtb*-infected young alcoholic mice is associated with the expression of molecules involved in necroptosis

**Fig 6:**
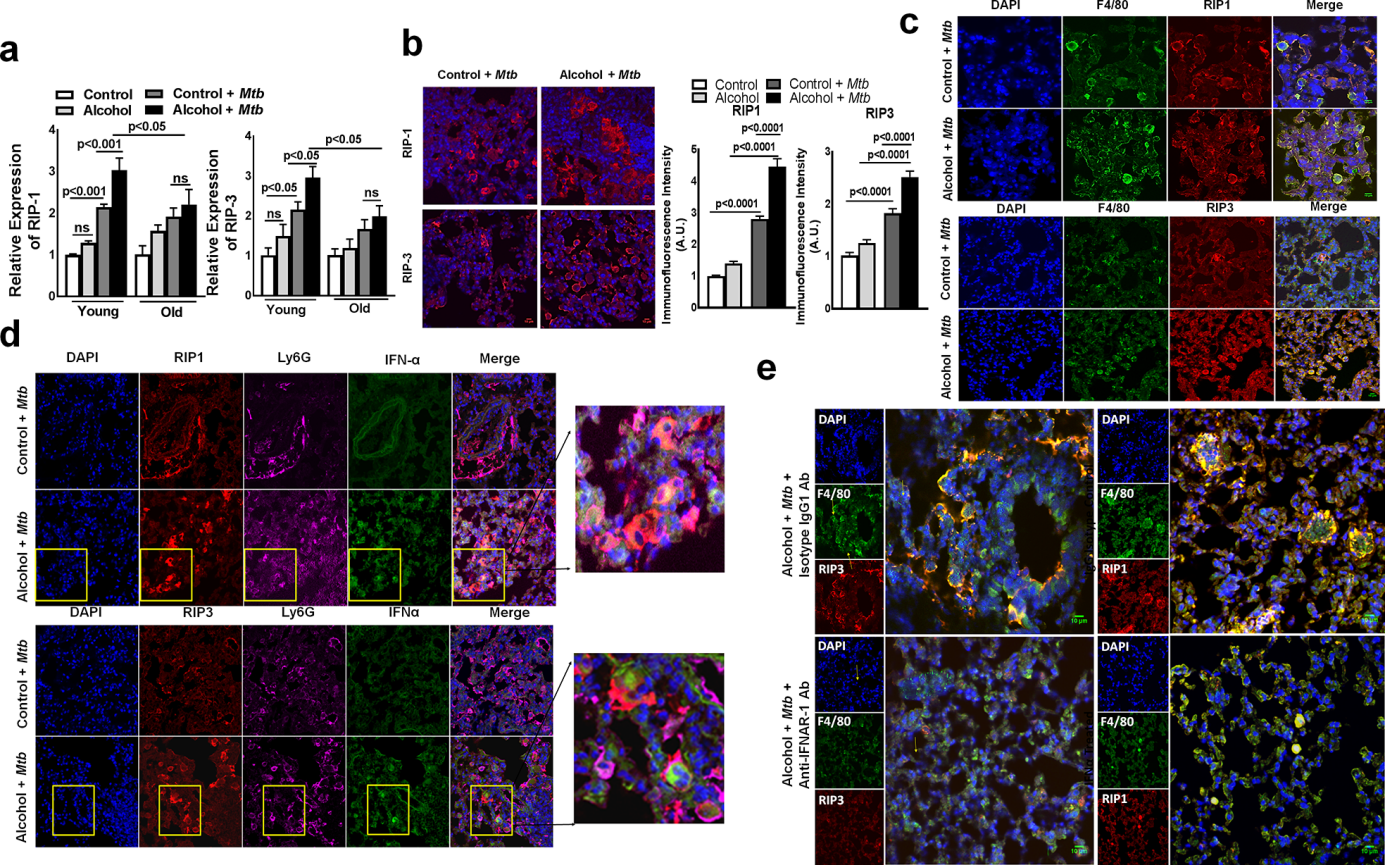
IFN-α production in *Mtb*-infected young alcoholic mice is associated with the expression of molecules involved in necroptosis. **a.** Young (one to two months of age) and old (17 to 22 months of age) mice were fed control and alcohol diets for one month as detailed in the methods section; then, they were infected with 50–100 CFU of aerosolized *Mtb* H37Rv, and control and alcohol diet feeding was continued. At six months p.i., lung homogenates from uninfected control and alcohol diet-fed mice and *Mtb*-infected control and alcohol diet-fed mice were collected, and RIP-1 and RIP-3 gene expression was determined by quantitative real-time PCR. Control and alcohol diet-fed young mice were infected with 50–100 CFU of aerosolized *Mtb*. At three months p.i., lungs from uninfected control and alcohol diet-fed mice as well as from *Mtb*-infected control and *Mtb*-infected alcohol diet-fed mice were isolated and formalin fixed. Paraffin-embedded tissue sections were prepared and analyzed by confocal microscopy for **b**. RIP-1+ and RIP-3+ cells (red). Representative images of staining patterns were taken of multiple fields at 40X and 63X with oil immersion. The immunofluorescence intensities for these groups were calculated. **c.** RIP-1- and RIP-3 (red)-expressing F4/80 macrophages (green). **d.** Lung paraffin-embedded tissue sections were analyzed by confocal microscopy to determine IFN-α (Green), Ly6G (Magenta or Far-red) and RIP1/3 (red) colocalization. Scale bar: 10 μm. The rightmost panel shows a higher magnification for representation, and the yellow squares represent IFN-α-expressing Ly6G*+* cells. **e.** Representative images of RIP-1 and RIP-3 expression in lung F4/80 macrophages from anti-IFNAR-1 antibody-treated and isotype control antibody (IgG1)-treated mice are shown. Five mice per group were used for each group. The mean values, p-values and SEs are shown.

We further examined the lung lesions of *Mtb*-infected (three months after infection) alcohol diet-fed young mice using confocal microscopy. As shown in S3 Fig, cleaved caspase 3 expression was similar in *Mtb*-infected young alcoholic mice, *Mtb*-infected control and uninfected alcoholic mice, suggesting that there is no significant difference in lung cell apoptosis in these groups of mice. We next examined the expression of receptor-interacting serine/threonine-protein kinase (RIP)-1 and RIP-3, which are known to be expressed by cells undergoing programmed necrotic cell death (necroptosis) [29]. Six months after *Mtb* infection, lungs were isolated from control and alcohol diet-fed young and old mice, and the gene expression levels of RIP-1 and RIP-3 were determined by real-time PCR. As shown in Fig 6A, the expression levels of RIP-1 and RIP-3 in the lungs were significantly higher in *Mtb*-infected alcohol diet-fed young mice than in *Mtb*-infected alcohol diet-fed old mice and control diet-fed young mice. Confocal microscopy examinations of the lung sections also indicated significantly higher RIP-1 and RIP-3 expression in the lung lesions of *Mtb*-infected alcohol diet-fed young mice than in the lungs of *Mtb*-infected control and uninfected alcoholic mice (Fig 6B and S3B Fig). RIP-1 and RIP-3 are expressed by F4/80 macrophages but were not expressed by IFN-α-producing Ly6G*+* cells (Fig 6C and 6D). To determine whether IFN-α-producing Ly6G*+* cells are involved in the enhanced RIP-1 and RIP-3 expression in lung macrophages, we first used confocal microscopy to examine the lung sections of *Mtb*-infected young alcoholic mice for Ly6G*+* and RIP-1 and RIP-3-expressing F4/80+ cell interactions. At three months p.i., the imaging results indicated that RIP-1 and RIP-3-expressing F4/80+ cells from *Mtb*-infected young alcoholic mice were in closer proximity to IFN-α-producing Ly6G*+* cells than those from *Mtb*-infected control mice (Fig 6D and S4 Fig). More importantly, our results indicate that the marked increase in IFN-α production was spatially defined at the region where both Ly6G*+* and RIP-1 and RIP-3-expressing F4/80+ cells interact with each other (Fig 6D). RIP-1 and RIP-3 expression levels in the lungs were significantly lower in anti-IFNAR-1 mAb-treated *Mtb*-infected young alcoholic mice than in isotype antibody-treated *Mtb*-infected young alcoholic mice (Fig 6E).

### Alcohol does not enhance IFN-*α* production in old mice infected with *Mtb*

We compared IFN-α production in the lungs of alcohol-fed young and old mice following *Mtb* infection. Young and old mice were fed control and alcohol diets and infected with *Mtb* as in Fig 1. After three months, the mice were euthanized, and the lung sections were examined for IFN-α by confocal microscopy. As shown in S5 Fig, the immunofluorescence intensity for IFN-α was significantly lower in *Mtb*-infected old alcoholic mice than in *Mtb*-infected young alcoholic mice. We also found that RIP-1 and RIP-3 expression levels were significantly lower in the *Mtb*-infected old alcoholic mice than in the *Mtb*-infected young alcoholic mice (S5B, S5C and S5D Fig).

### Alcoholism enhances IFN-*α* production by PBMCs from healthy young LTBI+ individuals

**Fig 7:**
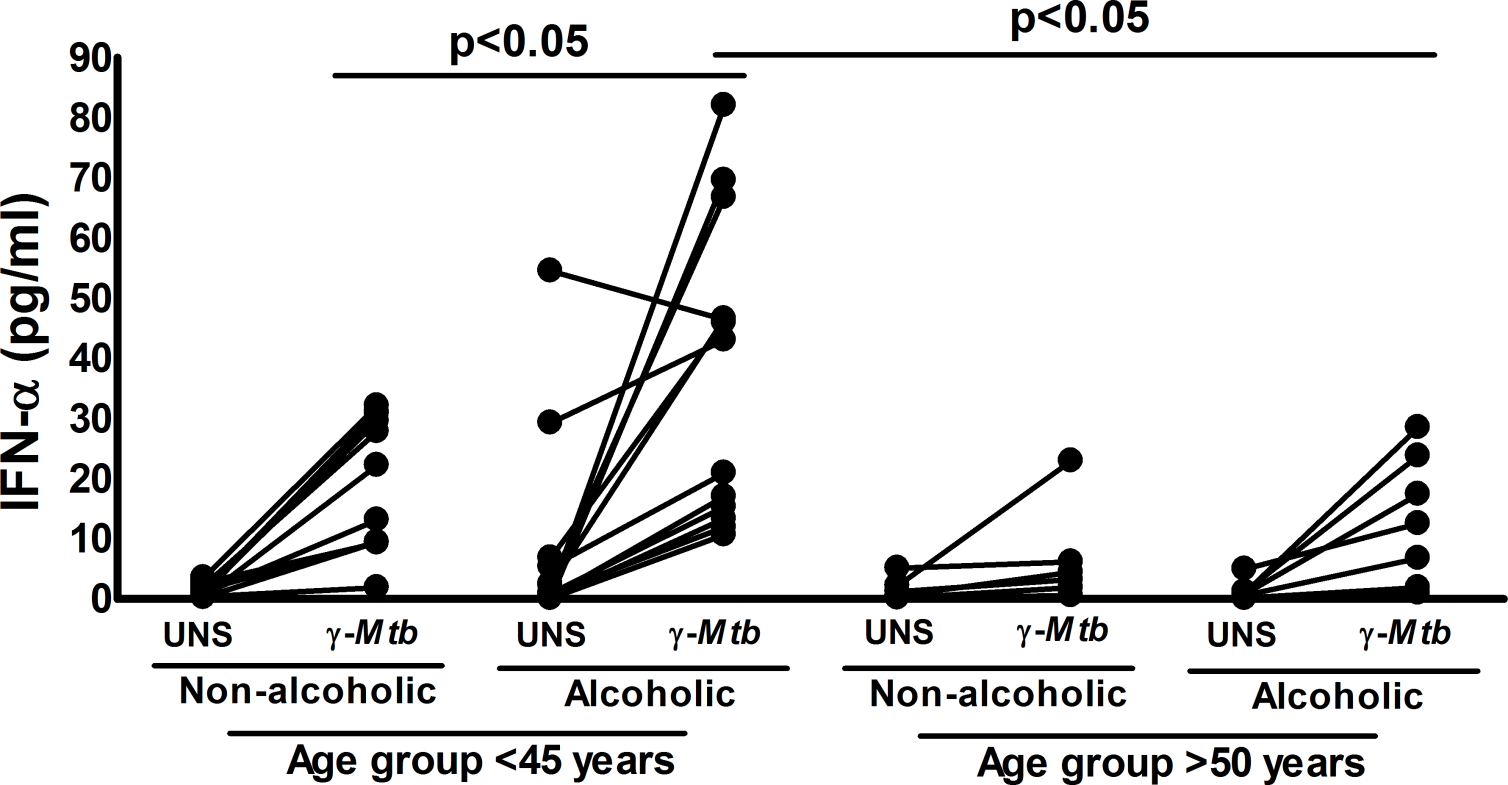
Alcoholism enhances *Mtb*-induced IFN-α production by PBMCs from young LTBI+ individuals. Blood samples were obtained from 17 non-alcoholic and 20 alcoholic pulmonary tuberculosis patients who were 18–75 years of age. On the basis of age, LTBI+ individuals were categorized in to young (n = 24, age <45) and old (n = 11, age >50 years) groups. PBMCs from alcoholic and non-alcoholic young and old LTBI+ individuals were cultured with or without γ-irradiated *Mtb* (10 μg/ml). The levels of IFN-α were determined by ELISA. The mean values, p-values and SEs are shown.

To determine the relevance of the above findings to the clinical manifestation of human *Mtb* infection, we obtained blood samples from alcoholic and non-alcoholic LTBI+ individuals. First, on the basis of age, we characterized the LTBI+ individuals by age group: <45 years (young) and >50 years (old). We cultured peripheral blood mononuclear cells (PBMCs) in the presence of 10 μg/ml γ-irradiated *Mtb*. After 72 hours, IFN-α levels were determined by ELISA as detailed in the methods section. As shown in Fig 7, γ-irradiated *Mtb* significantly enhanced the IFN-α levels by 2-fold in PBMCs from young alcoholic LTBI+ individuals and compared with those from non-alcoholic young LTBI+ individuals and by 2.9-fold compared to those from old alcoholic LTBI+ individuals. The baseline IFN-α levels were also high in young alcoholic LTBI+ individuals compared with those of other groups (Fig 7).

## Discussion

Chronic alcohol consumption modulates host immune defense mechanism(s) and makes the host susceptible to various fungal, viral and bacterial infections, including *Mtb* [13,15,19]. However, limited information is available regarding the mechanisms involved in alcohol-mediated host susceptibility to *Mtb* and other intracellular bacterial infections. In the current study, we fed young and old mice control and alcohol diets and determined the mortality rates and the immune mechanisms involved in host susceptibility to *Mtb* infection. Approximately 80% of the *Mtb*-infected alcohol-fed young mice died within 5 months; however, only 25% of *Mtb*-infected alcohol-fed old mice and 25% of alcohol-fed uninfected young mice died during the same period. There were no significant differences in the bacterial lung burdens of control and alcohol diet-fed young mice and alcohol diet-fed old and young mice. IFN-α levels were significantly higher in the lungs of *Mtb*-infected alcohol-fed young mice, and treatment with an anti-IFNAR-1 antibody increased their survival. In the lungs of *Mtb*-infected alcohol-fed young mice, IFN-α enhanced the expression of RIP-1 and RIP-3, which are known to be involved in necroptosis. *Mtb*-infected alcohol-fed old mice and *Mtb*-infected control diet-fed old and young mice did not express IFN-α, RIP-1 or RIP-3 in their lungs. In response to *Mtb*, PBMCs from alcoholic LTBI+ healthy individuals produced significantly higher amounts of IFN-α than PBMCs from non-alcoholic young LTBI+ individuals and alcoholic and non-alcoholic aged LTBI+ individuals. Our findings demonstrate that alcohol enhances Ly6G+ cell infiltration and IFN-α production and increases necroptosis in the lung macrophages of young mice infected with *Mtb*, which leads to enhanced mortality.

Chronic alcohol consumption inhibits host protective immune responses to infections, including *Mtb* infection, and increases the mortality rates of young and aged individuals [30,31]. According to the Centers for Disease Control’s (CDC) estimations, one-third of binge drinkers are old individuals, and human studies have found that compared young individuals, old individuals are more susceptible to various diseases [32–34]. Old individuals are likely to take prescribed medications, and in some cases, malnourishment and alcohol may have different effects on these individuals [34–36]. No experimental animal studies have been performed to determine the effects of chronic alcohol feeding in aging and *Mtb* infection. In the current study, we found that alcohol diet-fed young mice (1–2 months) are more susceptible to *Mtb* infection and have a higher mortality rate than alcohol diet-fed old mice (17–22 months) (Fig 1A). Our findings suggest that alcohol worsens the TB pathology in the early stage of life and leads to increased mortality.

We found that IFN-α is responsible for the early death of alcoholic *Mtb*-infected young mice. IFN-α is a type 1 interferon that belongs to the interferon family, which regulates the immune responses to infection, cancer and autoimmune diseases [37,38]. Type 1 interferons have a protective role during viral infections, but during *Mtb* infection, they enhance the pathogenicity [39,40]. *Mtb* proteins induce the production of type 1 interferons by host myeloid cells [41]. Type 1 interferons inhibit IL-1β production and enhance *Mtb* growth in myeloid cells [42]. In the current study, we found that IFN-α produced by Ly6G+ cells was associated with macrophage necroptosis and fatal immunopathology in the lungs of young alcohol diet-fed mice IFNAR1 signaling is detrimental during *Mtb* infection and promotes excess inflammation [28]. Furthermore, in the current study, we found that *Mtb*-infected alcohol diet-fed mice survived for a longer period of time and had less bacterial burden in their lungs when type 1 IFN signaling pathways were blocked.

Old mice express transient early resistance to pulmonary tuberculosis, and type 1 cytokines have no influence on this early resistance [43]. It is known that several signaling pathways are defective in old mice [44]. The transient resistance in *Mtb*-infected old mice is due to a population of memory CD8+ T cells that express several receptors for Th1 cytokines; in addition, in aged mice, lung macrophages secret more proinflammatory cytokines in response to *Mtb* [43]. We have not determined the CD8+ cell and macrophage responses in alcoholic old mice; however, our results demonstrate that alcohol-fed mice were unable to enhance IFN-α production in *Mtb*-infected old mice, and there were no effects on mortality compared to the non-alcoholic *Mtb*-infected old mice. IFN-α production in young alcoholic *Mtb*-infected mice significantly reduced their survival. Our current findings suggest that defective signaling pathways that are involved in the production of IFN-α in *Mtb*-infected old mice may be protecting them from alcohol-mediated lung cell necroptosis.

In various experimental models, it was shown that immune cells, such as macrophages, dendritic cells, T cells and Ly6G+ cells, produce type 1 interferons, and plasmacytoid dendritic cells are the major source [45]. However, only less than 1% of leucocytes are dendritic cells, and fifty percent of blood leucocytes are Ly6G+ cells [46]. Necrosis and Ly6G+ cell infiltration in the lung granuloma are characteristic features of tuberculosis granulomas, and these properties are associated with increased mycobacterial load and exacerbated lung pathology in human and experimental animals [47]. Netting Ly6G+ cells are the major inducers of type I IFN production [48]. Whole blood transcript signatures for active TB patients and pathway analyses revealed that the TB signature is dominated by a neutrophil-driven interferon (IFN)-inducible gene profile that consists of both IFN-γ and type I IFNαβ signaling [49]. Alcohol consumption can reduce the recruitment of Ly6G+ cells to the site of infection [19,50]. We have further investigated whether IFN-α-producing CD11b+ Ly6G+ cells are neutrophils. We found that these cells express a unique phenotype, including some neutrophils markers (positive for CD11b, CD200 and CD163 but negative for F4/80, CD68, CD115, CD11c, and Ly6C), suggesting these cells are not neutrophils. In the current study, we found fewer CD11b+Ly6G+ cells in the lungs of alcohol-fed *Mtb*-infected old mice than in those of alcohol-fed *Mtb*-infected young mice. We have not determined the factors underlying the reduced CD11b+Ly6G+ infiltration in the lungs of alcohol-fed *Mtb*-infected old mice, but it is known that in old individuals, the neutrophil lifespan is decreased, neutrophil precursors in the bone marrow proliferate less, neutrophil recruitment at the site of inflammation is reduced, and CD11b+ Ly6G+ cells are less functional due to alterations in signaling pathways [51,52]. Our results suggest that alcohol consumption can enhance these defects in old mice and that Ly6G+ neutrophil- like cells are unable to migrate to the lungs of *Mtb*-infected mice, resulting in less IFN-α production and necroptosis and enhanced survival.

We found that IFN-α produced by Ly6G+ cells in alcohol-fed *Mtb*-infected young mice induces necroptosis in lung macrophages. Necroptosis is programmed necrosis that differs from other death pathways (apoptosis, autophagy and pyroptosis) due to the requirement of a unique signaling pathway associated with the activation of receptor-interacting protein (RIP) kinases 1 and 3 [53,54]. Caspase 1 expression was similar among all groups of infected mice, but RIP-1 and RIP-3 expression was significantly higher in the lungs of alcohol-fed *Mtb*-infected young mice than in those of control diet-fed *Mtb*-infected young mice and alcohol diet-fed *Mtb*-infected old mice. We also found that RIP-1 and RIP-3 expression was restricted to macrophages and that IFN-α-producing Ly6G+ cells were colocalized around macrophages. These findings suggest that IFN-α-producing Ly6G+ cells in alcohol-fed *Mtb*-infected young mice enhance necroptosis in lung macrophages.

Necroptosis exacerbates inflammatory responses to infection which contributes to tissue damage and pathology [55,56]. Our current findings demonstrate that alcohol enhances IFN-α mediated necroptotic death of lung macrophages in young *Mtb*-infected mice (Fig 6C). This leads to tissue damage and mortality in young alcoholic *Mtb*-infected mice.

To determine the clinical relevance of our mouse studies, we compared IFN-α levels in the culture supernatants of γ-irradiated *Mtb*-cultured PBMCs from young and old alcoholic and non-alcoholic healthy LTBI+ individuals. We found that PBMCs from young alcoholic LTBI+ individuals produced significantly higher amounts of IFN-α after culture with γ-irradiated *Mtb* than those from young non-alcoholic, old alcoholic and old non-alcoholic healthy LTBI+ individuals (Fig 7). Our findings suggest that young alcoholic LTBI+ individuals have a higher risk of developing active TB infection.

In conclusion, our studies demonstrate that alcohol increases the mortality of young but not old mice infected with *Mtb*. The increased mortality of alcohol-fed *Mtb*-infected young mice is due to IFN-α production by Ly6G+ cells. Further characterization of the exact phenotype of CD11b+ Ly6G+ cells and the delineation of the mechanisms through which alcohol enhances IFN-α production by Ly6G+ cells during *Mtb* infection will facilitate the development of therapies for alcoholic individuals with latent and active *Mtb*. Our findings may also be applicable to other intracellular pathogen infections.

## Materials and methods

### Animals

All animal studies were performed with specific pathogen-free, 6- to 8- week -old and 17- to 22-month-old male and female C57BL/6 mice (Jackson Laboratory and National Cancer Institute). The Institutional Animal Care and Use Committee of the University of Texas Health Science Center at Tyler approved the studies. The animal procedures involving the care and use of mice were conducted in accordance with the guidelines of the NIH/OLAW (Office of Laboratory Animal Welfare).

### Blood donors

Blood was obtained from 17 non-alcoholic and 20 alcoholic healthy LTBI+ individuals who were 18–75 years of age. PBMCs were isolated from freshly collected blood samples. All subjects were HIV seronegative. The alcoholic LTBI+ individuals had a history of drinking at least 10–12 drinks per week.

### Ethics statement

All human studies were approved by the Institutional Review Board of the Bhagwan Mahavir Medical Research Centre, and informed written consent was obtained from all participants. All human subjects involved in our study were adults. All animal studies were approved by the Institutional Animal Care and Use Committee of the University of Texas Health Science Center at Tyler (Protocol #554). All animal procedures involving the care and use of mice were undertaken in accordance with the guidelines of the NIH/OLAW (Office of Laboratory Animal Welfare).

### Chronic alcohol diet

All mice were maintained on a standard rodent chow diet (LabDiet, catalog number 5053, St. Louis, MO, 4.07 kcal/gm) until the beginning of the experiment, when they were randomized into control or alcohol-containing liquid diet groups. The mice were fed alcohol using the Lieber-DeCarli liquid diet formulation (Dyets Inc., catalog number 710260; Bethlehem, Pa.4.5 kcal/gm), which supplies 36% of the caloric intake as ethanol, or were fed an isocaloric liquid control diet (LCD) (Dyets Inc., catalog number 710027; Bethlehem, Pa, 4.5 kcal/gm) as previously described [57]. The animals were fed the respective liquid diets for 5 of 7 days and the chow diet for 2 of 7 days. Animals in the liquid ethanol diet (LED) group were given water containing 20% (wt/vol) ethanol on the two chow diet days. The weights of the mice were recorded weekly.

### Aerosol infection of mice with *Mtb* H37Rv

Mice were fed the alcohol and control diets, and after three months, they were infected with *Mtb* H37Rv using an aerosol exposure chamber as described previously [58]. Briefly, *Mtb* H37Rv was grown to the mid-log phase in liquid medium and then frozen in aliquots at -70°C. Bacterial counts were determined by plating on 7H10 agar supplemented with oleic albumin dextrose catalase (OADC). For infection, the bacterial stocks were diluted in 10 ml of normal saline (to 0.5 ×10^6^ CFU [colony forming units]/ml, 1 ×10^6^ CFU/ml, 2 ×10^6^ CFU/ml, and 4 × 10^6^ CFU/ml) and placed in a nebulizer within an aerosol exposure chamber custom made by the University of Wisconsin. In the preliminary studies, groups of three mice were exposed to the aerosol at each concentration for 15 min. After 24 h, the mice were euthanized, and homogenized lung samples were plated on 7H10 agar plates supplemented with OADC. CFUs were counted after 14–22 days of incubation at 37°C. The aerosol concentration that resulted in ~50–100 bacteria in the lungs was used for the subsequent studies.

### Anti-IFNAR-1 treatment

For some experiments, mice were treated with anti-IFNAR-1 antibodies. One month after control and alcohol diet feeding, the mice were challenged with aerosolized *Mtb*. After 3 months, the mice received 0.3 mg of anti-IFNAR-1 (BioXcell, Clone: MAR1-5A3, Catalog number: BP0241) or isotype-matched control Ab (rat IgG1, Clone: MOPC-21, Catalog number: BE0083) intravenously every 4 days for up to 2 months.

### Lung cell preparation

Lungs were harvested from the alcohol and control diet-fed mice at the indicated time points after *Mtb* challenge and were placed into 60-mm dishes containing 2 ml of Hank's balanced salt solution (HBSS). The tissues were minced with scissors into pieces no larger than 2–3 mm, and the fluid was discharged onto a 70-μm filter (BD Biosciences, San Jose, CA) that had been pre-wetted with 1 ml of PBS containing 0.5% bovine serum albumin (BSA, Sigma-Aldrich) suspended over a 50-ml conical tube. The syringe plunger was then used to gently disrupt the lung tissue before washing the filter with 2 ml of cold PBS/0.5% BSA. The total number of viable cells in the lungs was determined with the trypan blue exclusion method. For flow cytometry experiments, we gated based on the total lung CD45+ cells (leukocytes) and measured various cell populations.

### Abs and other reagents

For flow cytometry, we used FITC anti-CD3, PE anti-CD8, APC anti-CD4, APC anti-NK1.1, APC anti-CD11b, FITC anti-Ly6G, PE anti-IFN-α, APC CD11-C, and FITC anti-F4/80 antibodies (all from BioLegend). The antibodies used for the *in vivo* neutralization experiments were purchased from BioXcell (anti-mouse IFNAR-1, Clone: MAR1-5A3, Catalog number: BP0241, and mouse IgG1 isotype control, Clone: MOPC-21, Catalog number: BE0083). Anti-Ly6G (Sigma-Aldrich; MABF474), anti-F4/80 (Abcam; ab6640), anti-IFN-α-FITC conjugated (R&D Systems; 22100–3), anti-cleaved caspase-3 (Cell Signaling Technology; 9661S), anti-CD163 (Santa Cruz Biotechnology, INC; sc-58965), anti-Ly6C (Santa Cruz Biotechnology, INC; sc-52650), anti-CD115 (Santa Cruz Biotechnology, INC; sc-46662), anti-CD200 (Santa Cruz Biotechnology, INC; sc-53100), anti-CD11c (Abcam; ab33483), anti-CD68 (Abcam; ab53444), anti-RIP-1/3 (Santa Cruz Biotechnology, INC; sc-133102/sc-374639) and secondary antibodies (goat anti-rat IgG (H+L) -Alexa 647, goat anti-rabbit IgG (H+L), Alexa Fluor 488, and goat anti-mouse IgG (H+L), Alexa Fluor 594) were obtained from Life Technologies, and fluoroshield mounting medium with DAPI (Abcam, ab104139) was used for the confocal microscopy analyses.

### Flow cytometry

For surface staining, 10^6^ cells were resuspended in 100 μl of staining buffer (PBS containing 2% heat-inactivated FBS) and Abs. The cells were then incubated at 4° C for 30 min, washed twice and fixed in 1% paraformaldehyde before acquisition using a FACS Calibur flow cytometer (BD Biosciences). In some experiments, intracellular staining for IFN-α was performed. Controls for each experiment included cells that were unstained, cells to which PE-conjugated rat IgG had been added and cells that were single stained, either for a surface marker or for intracellular molecules. For IFN-α analysis, we gated based on CD11c, F4/80, CD11b or Ly6G-positive cells and determined the percentages or the number of IFN-α expressing cells.

### Measurement of cytokine production by multiplex ELISA

In the lung homogenates, the following 27 cytokines and chemokines were measured using a multiplex ELISA kit (M60009RDPD, Bio-Rad). The cytokines and chemokines analyzed were IL-1b, IL-1ra, IL-2, IL-4, IL-5, IL-6, IL-7, IL-8, IL-9, IL-10, IL-12 (p70), IL-13, IL-15, IL-17, basic FGF, eotaxin, G-CSF, GM-CSF, IFN-γ, IP-10, MCP-1 (MCAF), MIP-1a, MIP-1b, PDGF-BB, RANTES, TNF-α and VEGF.

### Real-time PCR

RNA was isolated from lungs using TRIzol (Invitrogen) according to the manufacturer's instructions. Complementary DNA (cDNA) was generated from 0.5 mg of RNA and random hexamer primers using a Maxima First Strand cDNA Synthesis Kit for RT-qPCR (BIO-RAD) according to the manufacturer's instructions, and real-time PCR was performed. Gene expression for RIP-1 and RIP-3 was determined using Sybr green master mix (Qiagen), gene-specific primers (Sigma-Aldrich) and an ABI Prism 7600. All gene expression levels were normalized to β-actin internal controls, and the fold changes were calculated using the 2-ΔΔCT method.

### Measurement of IFN-*α*

IFN-α levels were measured using ELISA kits (Abcam, USA, catalog number: ab213479) according to the manufacturer’s instructions.

### Measurement of serum alcohol levels

Serum was collected without anti-coagulant by cardiac puncture from control and alcohol diet-fed mice. Serum alcohol levels were determined by using an ethanol assay kit as per the manufacturer’s guidelines (Abcam, USA, catalog number: ab65343).

### Histopathological assessment of necrotic lesions in the lung

At the specified time points, mice were euthanized, and the harvested lungs were placed in 10% neutral buffered formalin (Statlab, McKinney, TX, USA) for 48 hours to inactivate the infectious agent. Paraffin-embedded blocks were cut into 5 μm-thick sections. For morphometric lesion analyses, the lung sections were stained with hematoxylin and eosin (H&E) and examined in a blinded manner to assess the necrotic lesions as previously described by Sibila et al. [59]. Briefly, each lung lobe was quantified for the lesion area and percentage of the lung lesions by using digital software (NIH ImageJ; developed at the U.S. National Institutes of Health and available on the Internet at https://imagej.nih.gov/ij/). Two investigators, DT and SC, independently assessed the immunohistochemical readouts using morphometric analyses.

### Confocal microscopy

Confocal microscopy was performed to colocalize IFN-α-producing Ly6G and RIP1/3-expressing F4/80 cells in the lung sections. The lung tissues were stored in 10% neutral buffered formalin; then, the samples were paraffin embedded and cut into 5 μM thick sections that were deparaffinized and rehydrated. The tissue sections were subjected to heat-induced antigen retrieval in 10 mM sodium citrate buffer (pH 6.0). Then, the lung tissue sections were incubated in 0.025% Triton X-100 in PBST for 10 min and washed 3 × 5 min using PBS. Nonspecific binding was blocked with 5% goat serum in PBST for 1 hour, and the slides were washed 2 × 5 min with PBS. The slides were then incubated at 4°C overnight in PBST with the appropriate dilutions of the following primary antibodies: anti-Ly6G (1:200), anti-F4/80 (1:50), anti-IFN-α-FITC-conjugated (1:50), anti-cleaved caspase-3 (1:400), anti-CD68 (1:100), anti-CD115 (1:50), anti-CD200 (1:50), anti-CD163 (1:50), anti-CD11c (1:100), anti-Ly6C (1:50) and anti-RIP-1/RIP-3 (1:50); subsequently, the slides were washed thoroughly 3 × 5 min with PBS. Then, the tissue sections were stained with the respective secondary antibodies at 1:1000 dilutions (v/v), washed again with PBS for 3 × 5 min, and mounted with fluoroshield mounting medium with DAPI. The slides were then examined and analyzed under a laser-scanning confocal microscope (Zeiss LSM 510 Meta). An IgG isotype secondary control was used for all the confocal microscopy studies, and Zen 2009 software (Carl Zeiss) was used for image acquisition; then, the images were processed/quantified uniformly for each experiment using ImageJ NIH software. Representative images from three different independent experiments are shown.

### Statistical analysis

Data analyses were performed using GraphPad Prism (GraphPad Software, Inc., La Jolla, CA). The results are expressed as the mean ± SE. For normally distributed data, comparisons between groups were performed using a paired or unpaired t-test and ANOVA as appropriate. Mouse survival was compared using the Kaplan- Meier log-rank test.

## Supporting information

S1 Fig Serum alcohol and liver enzyme levels of young and old alcoholic mice infected with *Mtb*. Young (one to two months of age) and old (17 to 22 months of age) mice were fed control and alcohol diets as detailed in the methods section and were infected with 50–100 CFU of aerosolized *Mtb* H37Rv, and control and alcohol diet feeding was continued. **a.** At one month p.i., blood was obtained from uninfected control and alcohol diet-fed mice as well as from *Mtb*-infected control and *Mtb*-infected alcohol diet-fed mice; serum alcohol levels were measured, and **b.** liver enzyme alanine transaminase activity was determined. The data from two independent experiments were pooled. Three mice per group were used for each independent experiment (n = 6). The mean values, p-values and SEs are shown.
(TIF)

S2 Fig Absolute number of lung leukocyte populations in *Mtb*-infected alcohol and control diet-fed mice. **a and b.** Control and alcohol diet-fed mice were infected with 50–100 CFU of aerosolized *Mtb*. At three months p.i., the lungs from uninfected control and alcohol diet-fed mice and from *Mtb*-infected control and alcohol diet-fed mice were isolated. Representative flow cytometry figures showing various lymphocyte populations in the lungs of control, alcohol diet-fed, infected control and infected alcohol diet-fed mice are shown. This is a representative figure for Fig 3. The data are representative of two independent experiments. Five mice per group were used for each independent experiment.
(TIF)

S3 Fig Cleaved-caspase 3, RIP-1 and RIP-3 expression in the lungs of *Mtb*-infected young alcoholic mice. Control and alcohol diet-fed young mice were infected with 50–100 CFU of aerosolized *Mtb*. At three months p.i., the lungs from uninfected control and alcohol diet-fed mice as well as from *Mtb*-infected control and *Mtb*-infected alcohol diet-fed mice were isolated and formalin fixed. Paraffin-embedded tissue sections were prepared and analyzed by confocal microscopy for **a**. cleaved-caspase 3 expression, and the immunofluorescence intensities for these groups were calculated. **b.** Representative images of RIP-1 and RIP-3 (red) staining for the mouse groups are shown. Representative staining pattern images for three independent experiments are shown. Five mice per group were used for each independent experiment. The mean values, p-values and SEs are shown.
(TIF)

S4 Fig IFN-α-producing Ly6G and RIP1/3-expressing F4/80 cells are in close proximity to each other in *Mtb*-infected alcohol diet-fed mice. Control and alcohol diet-fed young mice were infected with 50–100 CFU of aerosolized *Mtb*. At three months p.i., lungs from uninfected control and alcohol diet-fed mice as well as from *Mtb*-infected control and *Mtb*-infected alcohol diet-fed mice were isolated and formalin fixed. Paraffin-embedded lung tissue sections were analyzed by confocal microscopy to determine IFN-α (Green), Ly6G (Magenta or Far-red) and RIP1/3 (red) colocalization. The lowermost panel shows representative higher magnification images. Scale bar: 10 μm. Representative staining pattern images of three independent experiments are shown. Five mice per group were used for each independent experiment.
(TIF)

S5 Fig Alcohol does not enhance IFN-α production and RIP1/3 in old mice infected with *Mtb*. Young (one to two months of age) and old (17 to 22 months of age) mice were fed the control and alcohol diets and were infected with 50–100 CFU of aerosolized *Mtb* H37Rv. At three months p.i., lungs from uninfected control and alcohol diet-fed mice as well as from *Mtb*-infected control and *Mtb*-infected alcohol diet-fed mice were isolated and formalin fixed. Lung paraffin-embedded tissue sections were analyzed by confocal microscopy and immunofluorescence intensity for **a.** IFN-α and **b.** RIP-1. **c.** The amount of RIP-3 molecules was calculated. **d.** Paraffin-embedded lung tissue sections from the old mice were analyzed by confocal microscopy to determine IFN-α (Green), Ly6G (Magenta or Far-red) and RIP1/3 (red) colocalization. Scale bar: 10 μm. Representative staining pattern images of three independent experiments are shown. Five mice per group were used for each independent experiment. The mean values, p-values and SEs are shown.
(TIF)

S6 Fig Characterization of IFN-α producing CD11b+Ly6G+ cells in young alcoholic mice infected with *Mtb*. Young mice were fed the control and alcohol diets and infected with 50–100 CFU of aerosolized *Mtb* H37Rv as detailed in the methods section. At three months p.i., the lungs were isolated and formalin fixed. Lung paraffin-embedded tissue sections were analyzed by confocal microscopy, for CD11b, F4/80, CD68, CD115, CD200, CD163, CD11c, and Ly6C were determined. Representative staining pattern images of three independent experiments are shown. Five mice per group were used for each independent experiment.
(TIF)
