# Supplementary figures and images for: Alcohol enhances type 1 interferon-α production and mortality in young mice infected with Mycobacterium tuberculosis
Source: PLoS Pathog. 2018 Aug 2;14(8):e1007174. doi: 10.1371/journal.ppat.1007174 (PMC6072099; doi:10.1371/journal.ppat.1007174)

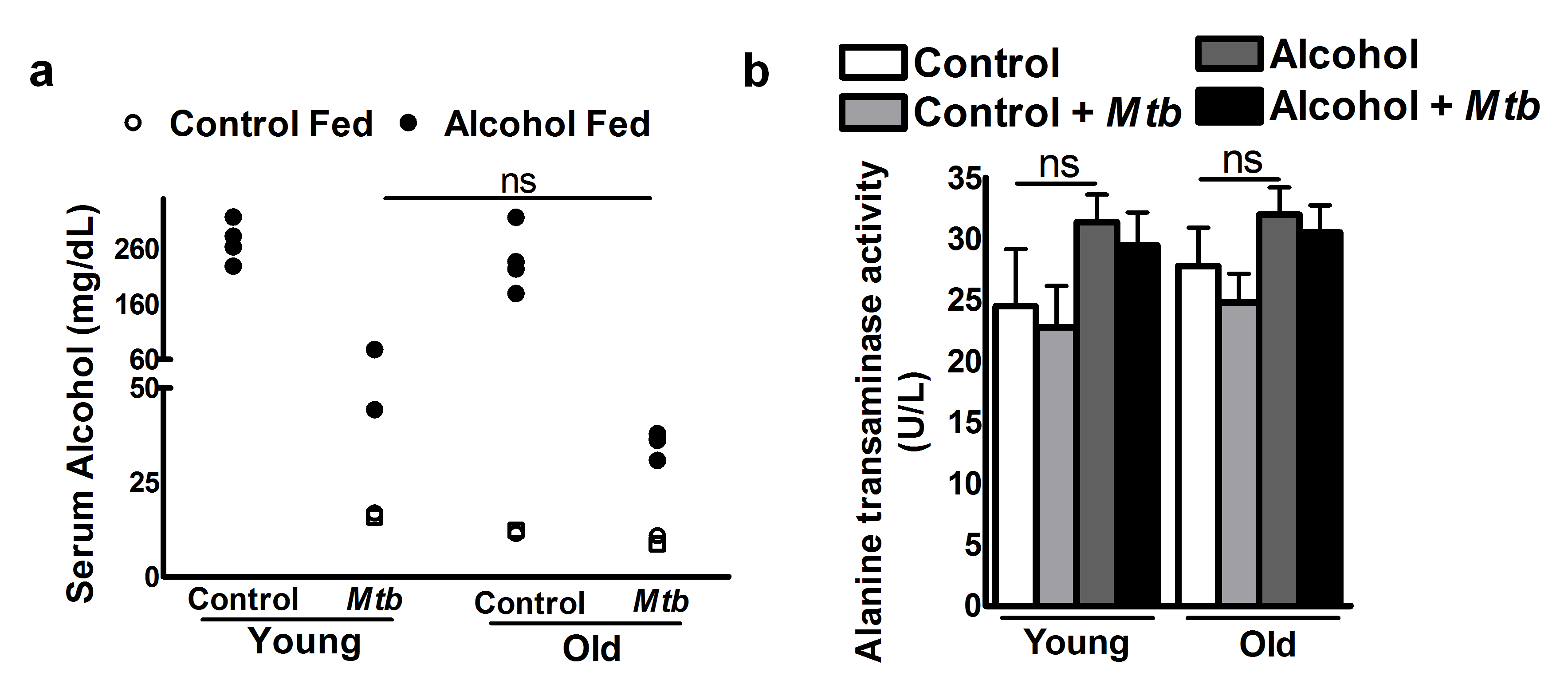

Supplement: S1 Fig — Young (one to two months of age) and old (17 to 22 months of age) mice were fed control and alcohol diets as detailed in the methods section and were infected with 50–100 CFU of aerosolized Mtb H37Rv, and control and alcohol diet feeding was continued. a. At one month p.i., blood was obtained from uninfected control and alcohol diet-fed mice as well as from Mtb-infected control and Mtb-infected alcohol diet-fed mice; serum alcohol levels were measured, and b. liver enzyme alanine transaminase activity was determined. The data from two independent experiments were pooled. Three mice per group were used for each independent experiment (n = 6). The mean values, p-values and SEs are shown. (TIF) [file ppat.1007174.s001.tif]

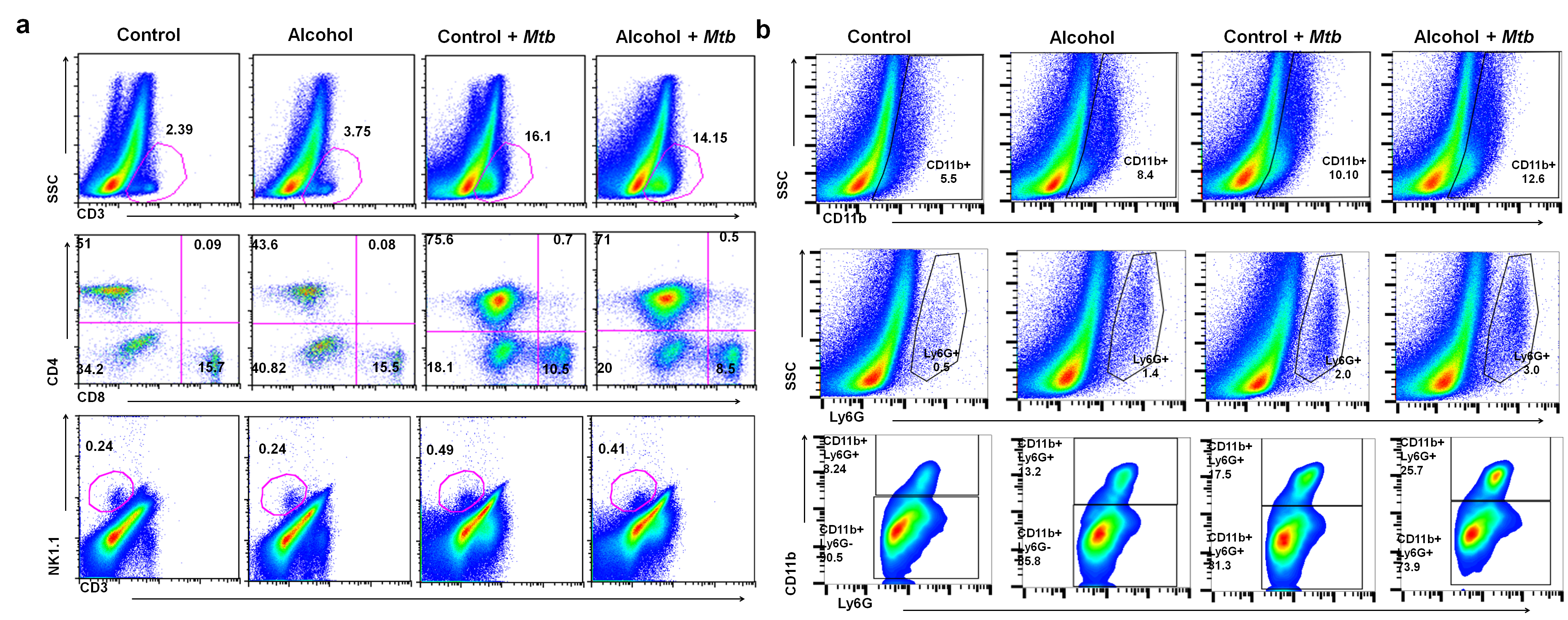

Supplement: S2 Fig — a and b. Control and alcohol diet-fed mice were infected with 50–100 CFU of aerosolized Mtb. At three months p.i., the lungs from uninfected control and alcohol diet-fed mice and from Mtb-infected control and alcohol diet-fed mice were isolated. Representative flow cytometry figures showing various lymphocyte populations in the lungs of control, alcohol diet-fed, infected control and infected alcohol diet-fed mice are shown. This is a representative figure for Fig 3. The data are representative of two independent experiments. Five mice per group were used for each independent experiment. (TIF) [file ppat.1007174.s002.tif]

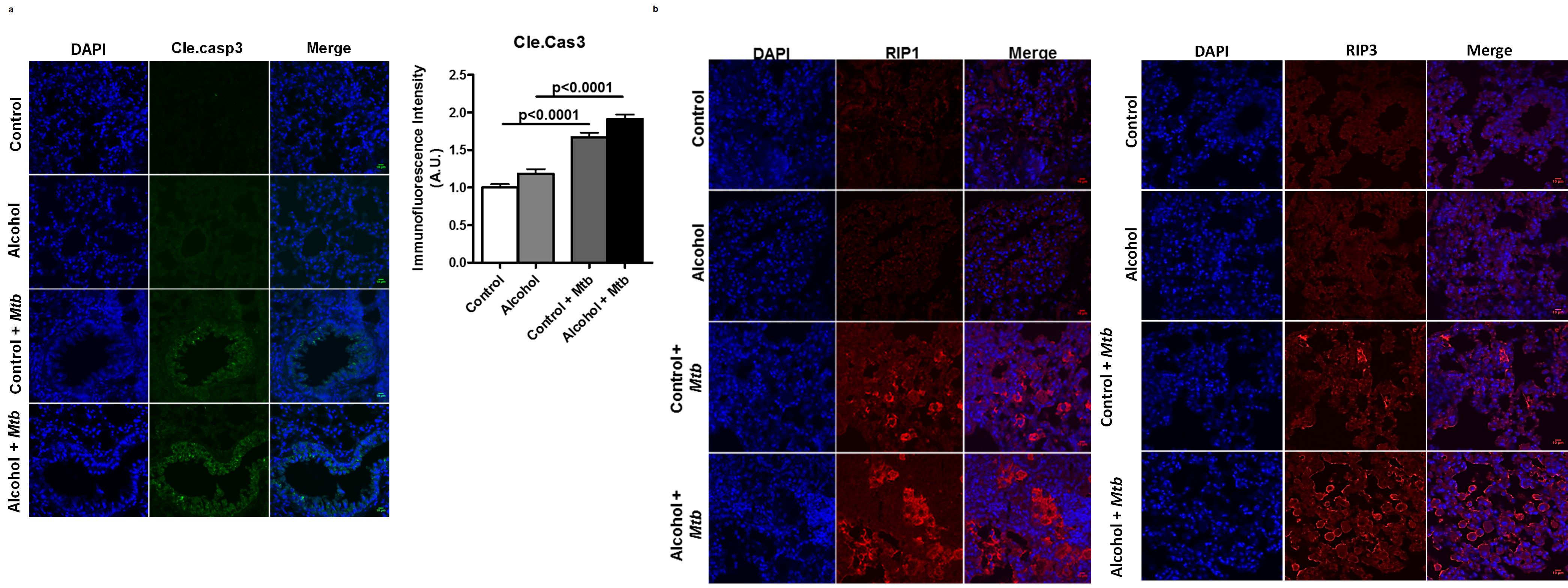

Supplement: S3 Fig — Control and alcohol diet-fed young mice were infected with 50–100 CFU of aerosolized Mtb. At three months p.i., the lungs from uninfected control and alcohol diet-fed mice as well as from Mtb-infected control and Mtb-infected alcohol diet-fed mice were isolated and formalin fixed. Paraffin-embedded tissue sections were prepared and analyzed by confocal microscopy for a. cleaved-caspase 3 expression, and the immunofluorescence intensities for these groups were calculated. b. Representative images of RIP-1 and RIP-3 (red) staining for the mouse groups are shown. Representative staining pattern images for three independent experiments are shown. Five mice per group were used for each independent experiment. The mean values, p-values and SEs are shown. (TIF) [file ppat.1007174.s003.tif]

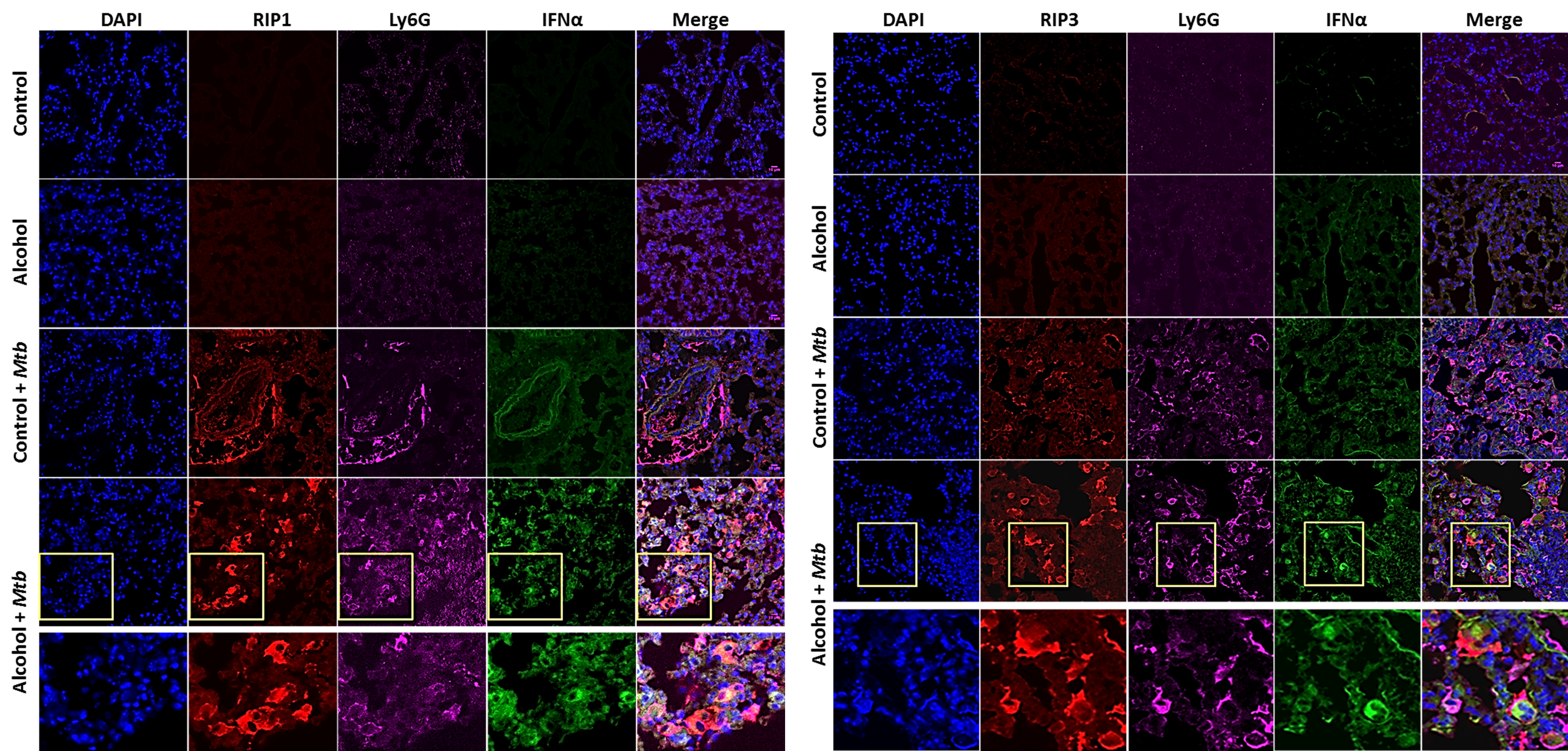

Supplement: S4 Fig — Control and alcohol diet-fed young mice were infected with 50–100 CFU of aerosolized Mtb. At three months p.i., lungs from uninfected control and alcohol diet-fed mice as well as from Mtb-infected control and Mtb-infected alcohol diet-fed mice were isolated and formalin fixed. Paraffin-embedded lung tissue sections were analyzed by confocal microscopy to determine IFN-α (Green), Ly6G (Magenta or Far-red) and RIP1/3 (red) colocalization. The lowermost panel shows representative higher magnification images. Scale bar: 10 μm. Representative staining pattern images of three independent experiments are shown. Five mice per group were used for each independent experiment. (TIF) [file ppat.1007174.s004.tif]

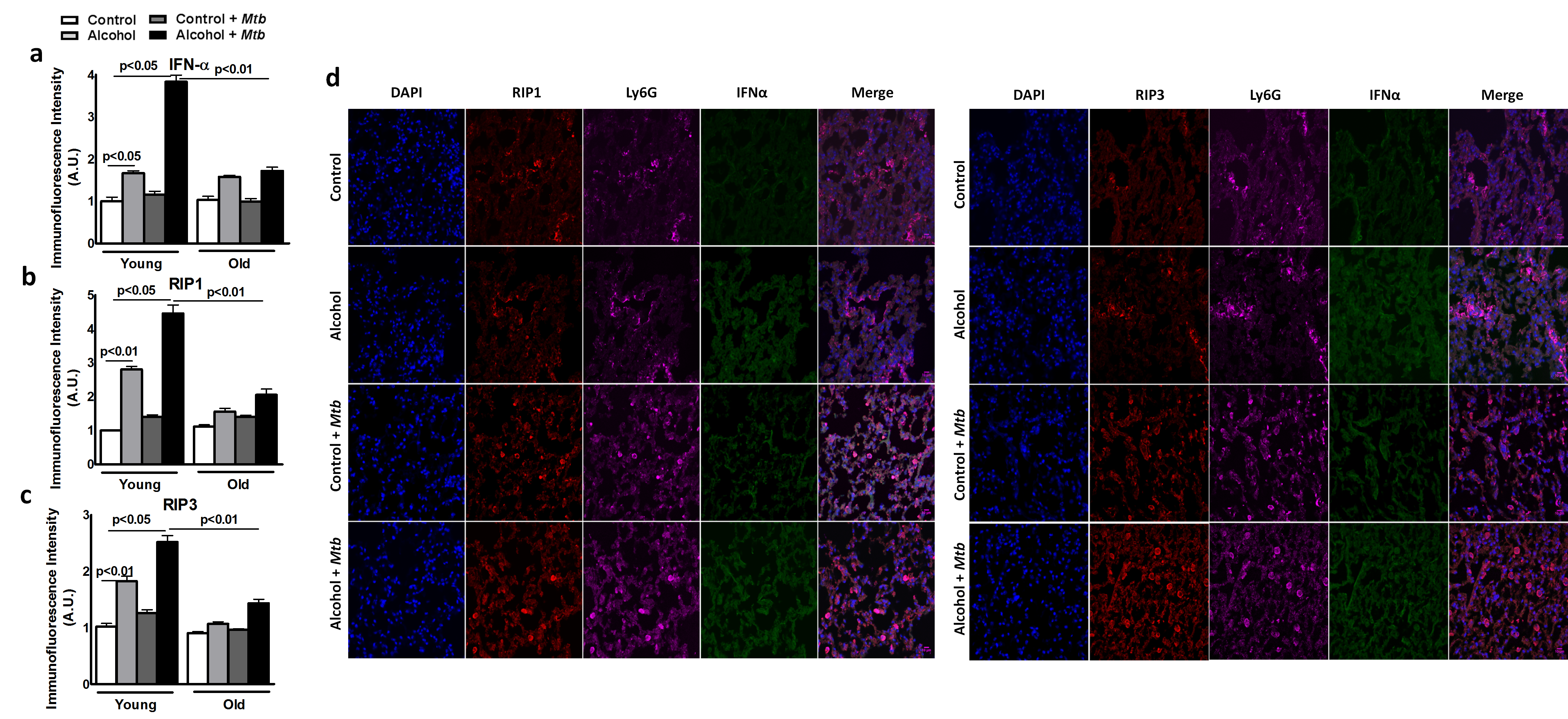

Supplement: S5 Fig — Young (one to two months of age) and old (17 to 22 months of age) mice were fed the control and alcohol diets and were infected with 50–100 CFU of aerosolized Mtb H37Rv. At three months p.i., lungs from uninfected control and alcohol diet-fed mice as well as from Mtb-infected control and Mtb-infected alcohol diet-fed mice were isolated and formalin fixed. Lung paraffin-embedded tissue sections were analyzed by confocal microscopy and immunofluorescence intensity for a. IFN-α and b. RIP-1. c. The amount of RIP-3 molecules was calculated. d. Paraffin-embedded lung tissue sections from the old mice were analyzed by confocal microscopy to determine IFN-α (Green), Ly6G (Magenta or Far-red) and RIP1/3 (red) colocalization. Scale bar: 10 μm. Representative staining pattern images of three independent experiments are shown. Five mice per group were used for each independent experiment. The mean values, p-values and SEs are shown. (TIF) [file ppat.1007174.s005.tif]

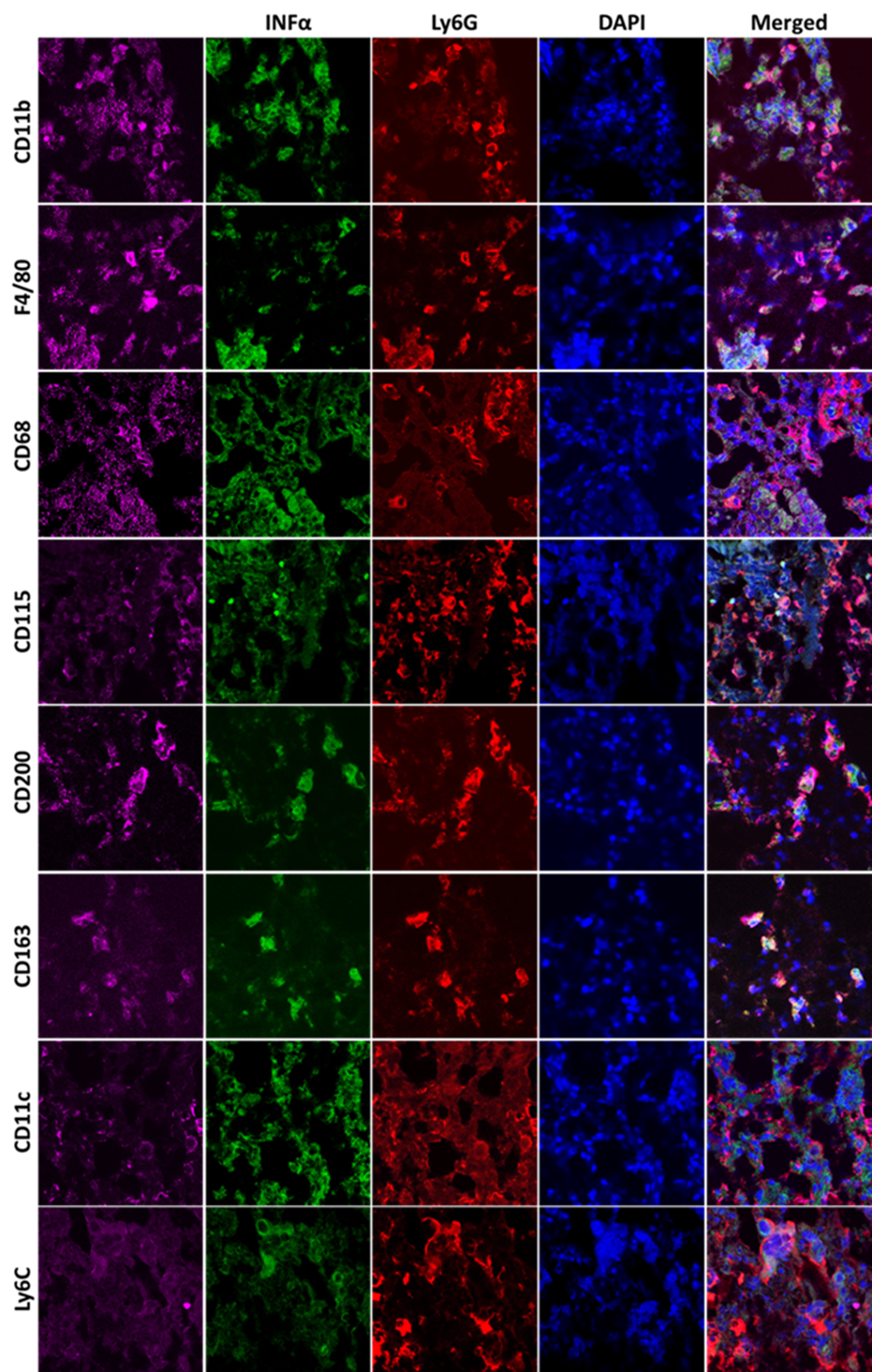

Supplement: S6 Fig — Young mice were fed the control and alcohol diets and infected with 50–100 CFU of aerosolized Mtb H37Rv as detailed in the methods section. At three months p.i., the lungs were isolated and formalin fixed. Lung paraffin-embedded tissue sections were analyzed by confocal microscopy, for CD11b, F4/80, CD68, CD115, CD200, CD163, CD11c, and Ly6C were determined. Representative staining pattern images of three independent experiments are shown. Five mice per group were used for each independent experiment. (TIF) [file ppat.1007174.s006.tif]
